# Effects of Bt Rice Straw Extract on Seed Germination and Plant Growth of Pakchoi: Novel Variables of Cropping System

**DOI:** 10.3390/plants14121797

**Published:** 2025-06-12

**Authors:** Chenning Zhang, Wenfang Suo, Yongfang Pan, Yuanjiao Feng

**Affiliations:** 1 Key Laboratory of Agro-Environment in the Tropics, Ministry of Agriculture and Rural Affairs, South China Agricultural University, Guangzhou 510642, China; zhangcn@stu.scau.edu.cn (C.Z.); swf1337846809@126.com (W.S.); panyf3721@163.com (Y.P.); 2 Guangdong Engineering Research Center for Modern Eco-Agriculture and Circular Agriculture, Guangzhou 510642, China; 3 Department of Ecology, College of Natural Resources and Environment, South China Agricultural University, Guangzhou 510642, China

**Keywords:** Bt rice, straw extract, pakchoi, seed germination, protective enzyme activity

## Abstract

The incorporation of *Bacillus thuringiensis* (Bt) rice straw into fields may influence the growth of subsequent crops, but its ecological risks for winter vegetables remain largely unreported. Investigating the effects of Bt rice straw extracts on the seed germination and plant growth of pakchoi (*Brassica campestris* L. ssp. *Chinensis* Makino var. *communis* Tsen et Lee) can provide a theoretical foundation for ecological risk assessments. In this study, straw extracts from non-Bt rice (Tianyouhuazhan), homozygous Bt rice (T775), and heterozygous Bt rice (F_1_ of T775 hybrid) were used as experimental materials at concentrations of 10, 20, and 40 g·L^−1^. Results showed that, compared to non-Bt extract, 40 g·L^−1^ homozygous Bt extract increased seedling height and leaf peroxidase (POD) activity but inhibited catalase (CAT) and root superoxide dismutase (SOD) activities. The 20 g·L^−1^ extract boosted root CAT activity yet suppressed leaf CAT and POD activities. The 10 g·L^−1^ extract enhanced root length but reduced leaf CAT and POD activities. The 40 g·L^−1^ heterozygous Bt extract increased leaf and root POD activity but inhibited germination rate and leaf SOD activity. The 10 g·L^−1^ extract promoted root length and seedling POD activity but suppressed leaf POD activity. In plant growth assessments, the 10 g·L^−1^ homozygous Bt extract reduced underground dry weight, and the 10 g·L^−1^ heterozygous Bt extract inhibited both above and underground dry weight, while the 20 g·L^−1^ heterozygous Bt extract increased aboveground dry weight. In conclusion, the effects of homozygous and heterozygous Bt rice straw extracts on pakchoi varied with concentration and physiological indices, showing no clear pattern. Optimizing straw return concentrations based on Bt rice variety differences is essential to mitigate ecological risks.

## 1. Introduction

Rice (*Oryza sativa* L.) serves as a principal staple crop, sustaining more than half of the global population, with its production being critically linked to worldwide food security. Statistical data indicate that annual global rice production is approximately 740 million metric tons, of which nearly 90% originates from Asian countries. This predominant cultivation in Asia underscores the region’s pivotal role in maintaining global rice supply chains and food security frameworks [1]. In China, rice is the main grain crop and plays a vital role in the country’s grain production sector. Improving rice varieties is key to ensuring the sustainable development of the seed industry and national food security [2]. However, lepidopterans and other pests are an important factor restricting rice production, and the pests such as borers and rice planthoppers have caused a 20–40% loss of global rice production, seriously threatening agricultural production [3].

The advent of transgenic technology has offered a groundbreaking approach to address the persistent challenge of developing insect-resistant rice varieties, revolutionizing modern crop improvement strategies. From 1996 to 2023, over 40 billion mu of GM crops have been planted globally [4]. Insecticidal proteins derived from Bt have been extensively utilized in transgenic crops to control lepidopteran pests [5]. Studies have reported that, in pesticide-free environments, transgenic Bt rice exhibits high resistance to pests and offers a significant yield advantage over non-Bt rice, with an increase of 9.1% to 38% [6]. Bt transgenic rice is the primary GM rice variety resistant to lepidopteran pests, with its insecticidal protein activating insecticidal activity by binding to specific receptors in target pests, leading to pest mortality while being harmless to humans [7,8,9,10,11]. However, despite the economic benefits associated with Bt rice cultivation, its potential ecological risks have attracted significant public concerns [2,12]. Despite demonstrating significant pest control efficacy in experimental trials, Bt rice has not yet been approved for commercial cultivation in the majority of countries, including China. Studies have shown that Bt proteins may enter the soil through root exudates or straw returning to the field, potentially affecting the structure and function of the soil microbial community [13]. The expression of Bt proteins may potentially affect ecosystems by modifying plant phenotypic traits (e.g., root exudate composition), regulating plant–organism interactions, and inducing trophic cascade effects. These potential ecological impacts require systematic evaluation through whole-plant experiments within environmental risk assessment (ERA) frameworks [14].

China is the world’s largest producer of agricultural straw, generating approximately 230 million tons annually [15]. In the Yangtze River rice-growing regions, the “rice–winter vegetable” rotation system predominates, where mechanized straw incorporation coupled with vegetable cultivation has become a key strategy for enhancing soil fertility. The direct return of rice straw to the field after harvest not only averts the loss of vital nutrients, including nitrogen, phosphorus, and potassium, during the decomposition process but also promotes the recycling of organic matter. Additionally, this practice alleviates pollution stemming from straw burning and improves soil fertility [16,17,18,19]. Notably, the October–November straw incorporation period overlaps with winter vegetable sowing, allowing early-stage straw leachates to directly interact with vegetable seedlings through soil solutions [20].

However, Bt rice straw contains Bt proteins and various bioactive allelopathic compounds, which may influence the growth of subsequent vegetable crops through the release of straw extracts [21,22,23]. Current research on the ecological risk assessment of Bt rice primarily focuses on its impact on non-target organisms in aboveground ecosystems [24,25,26,27,28,29], and the effects of Bt rice planting and straw incorporation on cropping remain relatively understudied [30,31,32,33]. While straw incorporation enhances soil fertility, it may also have potential implications for vegetable crop rotations. However, current risk assessments mainly focus on the direct effects of Bt crop cultivation, and there remains a lack of systematic studies on the long-term impacts of Bt rice straw incorporation on winter vegetable production.

Straw decomposition is a complex process influenced by multiple factors. In the early stages, straw initially undergoes a leaching phase, which gradually transitions into decomposition over time [34]. The extracts produced during straw decomposition can affect crop seed germination, induce physiological and biochemical responses in seedlings, and enhance their resistance to external stress. In laboratory experiments investigating the effects of straw incorporation on crop seedlings, the leaching method is widely used [35]. This method involves extracting straw-derived compounds and mixing them with natural soil to create a straw decomposition solution, simulating the decomposition process in the field and providing an experimental environment that closely mirrors real agricultural conditions.

Extensive studies have demonstrated that straw decomposition solutions regulate seed germination and influence seedling growth in various crops [36]. For instance, rice straw extract has been shown to promote the germination of sweet corn seeds, maintain the stability and integrity of plasma membranes, and enhance seedling growth and development. When rice straw is used as mulch in edamame fields, weed emergence rates can be reduced by approximately 20%, and weed seedling growth is significantly suppressed [37,38]. Yang et al. found that the water extract of thickened plum leaves exhibited an inhibitory effect on the seed germination of radish, pakchoi, and white birch [39]. Tang et al. reported that basil extract had a dual effect on little white seedlings, promoting seedling height, fresh weight, and dry weight at low concentrations while inhibiting them at higher concentrations [40]. Additionally, Tang et al. found that low concentrations of straw decomposition solution significantly reduced superoxide SOD and POD activities in buckwheat seedlings [36]. Li et al. observed that high concentrations of rice stem extract increased SOD and CAT activities in germinated wheat seeds 48 h after sowing [41]. Similarly, Xie et al. found that high concentrations of Torreya sinensis extract significantly elevated malondialdehyde (MDA) levels and the activities of antioxidant enzymes (SOD, CAT, and POD) in radish, mustard, and cabbage seedlings [42].

However, the impact of Bt rice straw extract on seed germination, protective enzyme activity, and the growth of pakchoi remains unclear. In this study, both non-Bt and Bt rice straw were used as experimental materials, and Bt rice straw extracts of varying concentrations and from different varieties were prepared. By comparing the effects of different Bt rice straw extracts, this study aims to provide a theoretical foundation for selecting Bt rice varieties that have minimal impact on pakchoi growth or even exhibit potential growth-promoting effects. Ultimately, the findings will contribute to a more comprehensive and scientifically sound basis for the rational and efficient utilization of Bt rice straw in agricultural production.

## 2. Results

### 2.1. Effects of Different Rice Straw Extracts on Seed Germination Rate and Seedling Growth of Pakchoi

Different rice straw extracts exhibited varying effects on the seed germination rate and seedling growth of pakchoi (Table 1). The seed germination rate of pakchoi exhibited a concentration-dependent decline, showing gradual decrease with increasing extract concentrations. Compared to non-Bt rice straw extracts, the 10 g·L^−1^ homozygous Bt rice straw extract significantly promoted root elongation in pakchoi seedlings, while the 40 g·L^−1^ homozygous Bt rice straw extract notably increased seedling height. Additionally, the 10 g·L^−1^ heterozygous Bt rice straw extract significantly enhanced root length, whereas the 40 g·L^−1^ heterozygous Bt rice straw extract significantly reduced the seed germination rate of pakchoi.

### 2.2. Effects of Different Rice Straw Extracts on Protective Enzyme Activities of Pakchoi Seedlings

#### 2.2.1. Effects on SOD Activity of Pakchoi Seedlings

**Figure 1:**
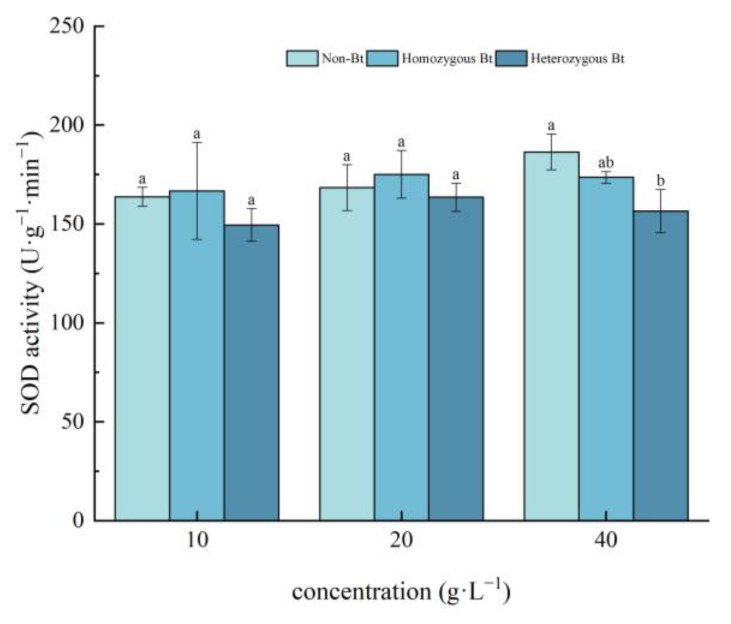
The effect of Bt rice straw extracts on SOD activity in the leaves of pakchoi seedlings. Values within the same column sharing the same letter indicate no significant difference (*p* > 0.05), whereas different letters denote a significant difference (*p* < 0.05). “Non-Bt” refers to the straw of non-Bt rice; “Homozygous Bt” refers to the straw of homozygous Bt rice; “Heterozygous Bt” refers to the straw of heterozygous Bt rice.

Different rice straw extracts had varying effects on SOD activity in the leaves and roots of pakchoi seedlings. As shown in Figure 1, compared to the non-Bt rice straw extract, the homozygous Bt rice straw extract at concentrations of 10, 20, and 40 g·L^−1^ showed no significant effect on SOD activity in pakchoi leaves. However, the 40 g·L^−1^ heterozygous Bt rice straw extract significantly inhibited SOD activity in the leaves, with an inhibition rate of 16.03%.

**Figure 2:**
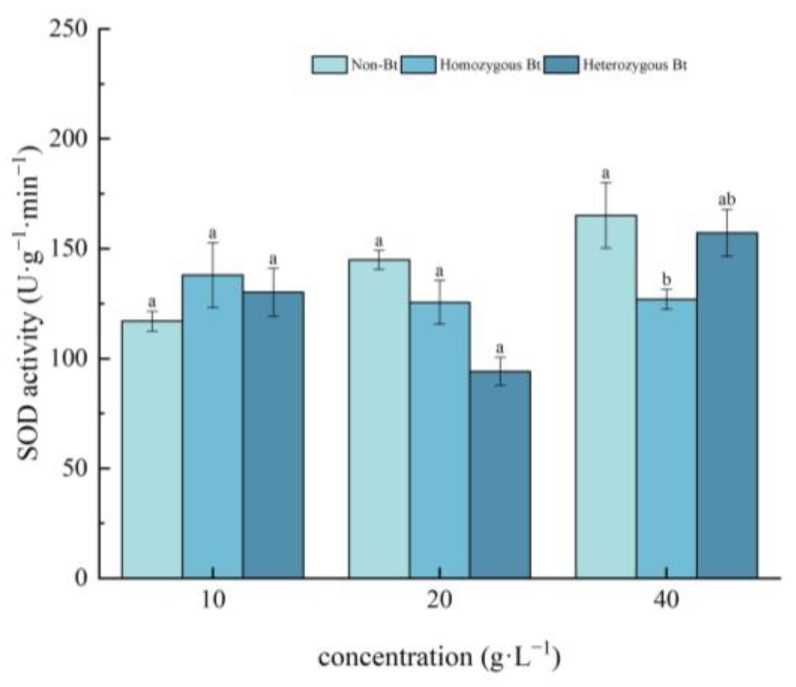
The effect of Bt rice straw extracts on SOD activity in the roots of pakchoi seedlings. Values within the same column sharing the same letter indicate no significant difference (*p* > 0.05), whereas different letters denote a significant difference (*p* < 0.05). “Non-Bt” refers to the straw of non-Bt rice; “Homozygous Bt” refers to the straw of homozygous Bt rice; “Heterozygous Bt” refers to the straw of heterozygous Bt rice.

As shown in Figure 2, compared to the non-Bt rice straw extract, the homozygous Bt rice straw extract at a concentration of 40 g·L^−1^ significantly suppressed SOD activity in the roots of pakchoi seedlings, with an inhibition rate of 23.13%.

#### 2.2.2. Effects on CAT Activity of Pakchoi Seedlings

**Figure 3:**
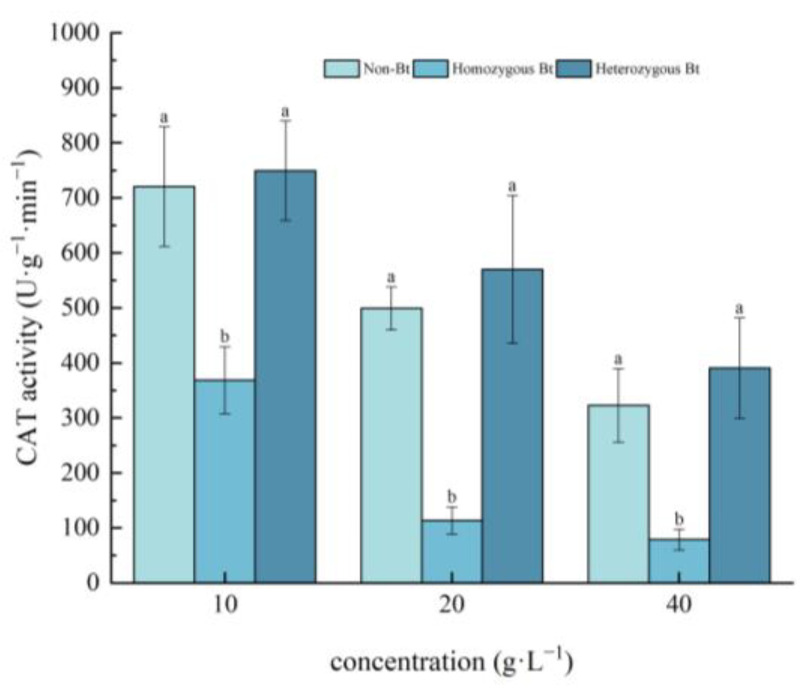
The effect of Bt rice straw extracts on CAT activity in the leaves of pakchoi seedlings. Values within the same column sharing the same letter indicate no significant difference (*p* > 0.05), whereas different letters denote a significant difference (*p* < 0.05). “Non-Bt” refers to the straw of non-Bt rice; “Homozygous Bt” refers to the straw of homozygous Bt rice; “Heterozygous Bt” refers to the straw of heterozygous Bt rice.

As shown in Figure 3, CAT activity in the leaves of pakchoi seedlings decreased with increasing extract concentration. Compared to the non-Bt rice straw extract, the homozygous Bt rice straw extract at concentrations of 10, 20, and 40 g·L^−1^ significantly inhibited CAT activity in the leaves, with inhibition rates of 48.86%, 77.29%, and 75.61%, respectively. In contrast, the heterozygous Bt rice straw extract had no significant effect on CAT activity in the leaves of pakchoi seedlings.

**Figure 4:**
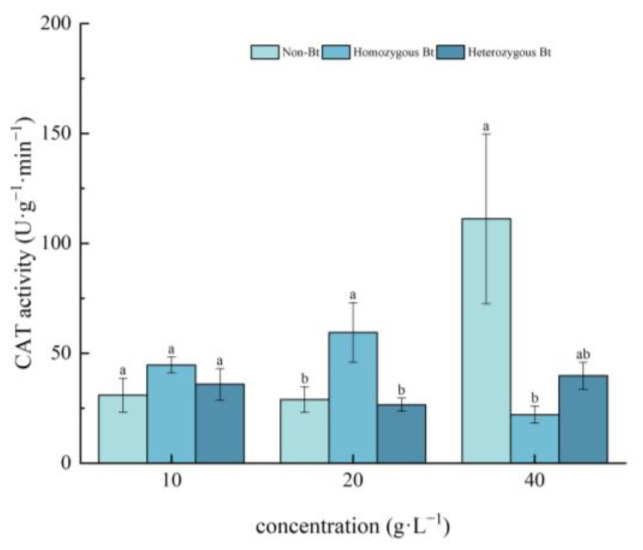
The effect of Bt rice straw extracts on CAT activity of pakchoi seedling roots. Values within the same column sharing the same letter indicate no significant difference (*p* > 0.05), whereas different letters denote a significant difference (*p* < 0.05). “Non-Bt” refers to the straw of non-Bt rice; “Homozygous Bt” refers to the straw of homozygous Bt rice; “Heterozygous Bt” refers to the straw of heterozygous Bt rice.

Different rice straw extracts had varying effects on CAT activity in the roots of pakchoi seedlings (Figure 4). Compared to the non-Bt rice straw extract, the homozygous Bt rice straw extract at 20 g·L^−1^ significantly enhanced CAT activity in the roots, with an increase of 51.29%. However, the homozygous Bt rice straw extract at 40 g·L^−1^ significantly inhibited root CAT activity, with an inhibition rate of 80.18%. In contrast, the heterozygous Bt rice straw extract had no significant effect on CAT activity in the roots of pakchoi seedlings.

#### 2.2.3. Effects on POD Activity of Pakchoi Seedlings

**Figure 5:**
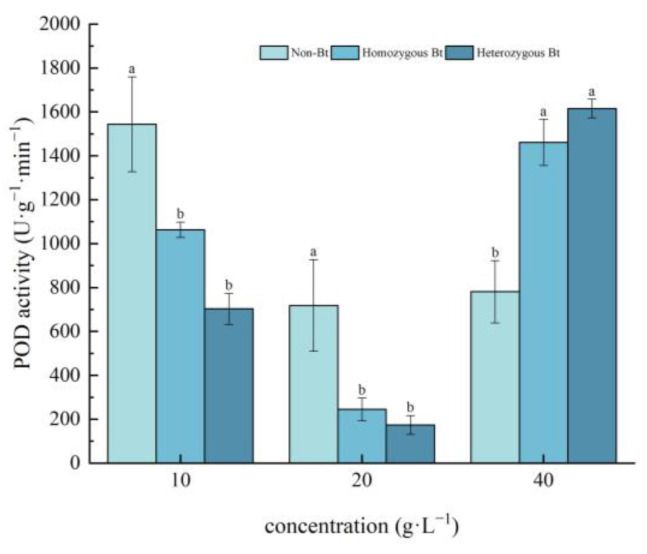
The effect of Bt rice straw extracts on POD activity in the leaves of pakchoi seedlings. Values within the same column sharing the same letter indicate no significant difference (*p* > 0.05), whereas different letters denote a significant difference (*p* < 0.05). “Non-Bt” refers to the straw of non-Bt rice; “Homozygous Bt” refers to the straw of homozygous Bt rice; “Heterozygous Bt” refers to the straw of heterozygous Bt rice.

**Figure 6:**
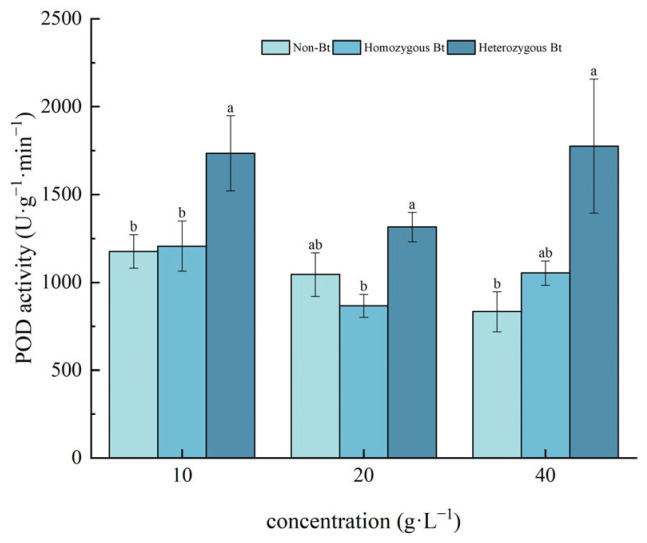
The effect of Bt rice straw extracts on POD activity of the roots of pakchoi seedlings. Values within the same column sharing the same letter indicate no significant difference (*p* > 0.05), whereas different letters denote a significant difference (*p* < 0.05). “Non-Bt” refers to the straw of non-Bt rice; “Homozygous Bt” refers to the straw of homozygous Bt rice; “Heterozygous Bt” refers to the straw of heterozygous Bt rice.

Different rice straw extracts had varying effects on POD activity in the leaves and roots of pakchoi seedlings (Figure 5 and Figure 6). As shown in Figure 5, compared to the non-Bt rice straw extract, the homozygous Bt rice straw extracts at 10 g·L^−1^ and 20 g·L^−1^ significantly inhibited POD activity in the leaves of pakchoi seedlings, with inhibition rates of 31.16% and 54.48%, respectively. However, the homozygous Bt rice straw extract at 40 g·L^−1^ significantly enhanced POD activity in the leaves, with an increase of 46.56%. Similarly, the heterozygous Bt rice straw extracts at 10 g·L^−1^ and 20 g·L^−1^ significantly suppressed POD activity in the leaves, with inhibition rates of 65.90% and 75.56%, respectively. In contrast, the 40 g·L^−1^ heterozygous Bt rice straw extract significantly increased POD activity in the leaves, with a growth rate of 51.66%.

As shown in Figure 6, compared to the non-Bt rice straw extract, the different concentrations of the homozygous Bt rice straw extract had no significant effect on POD activity in the roots of pakchoi seedlings. However, the heterozygous Bt rice straw extracts at 10 g·L^−1^ and 40 g·L^−1^ significantly increased POD activity in the roots, with growth rates of 32.21% and 53.07%, respectively.

### 2.3. Effects of Different Rice Straw Extracts on Growth of Pakchoi

The effects of the three types of rice straw extracts on the growth of pakchoi plants varied depending on the rice variety and extract concentration (Table 2). Compared to the non-Bt rice straw extract, different concentrations of both the homozygous and heterozygous Bt rice straw extracts had no significant effects on plant height or chlorophyll content in pakchoi. However, the homozygous Bt rice straw extract at 10 g·L^−1^ significantly inhibited the underground dry weight of pakchoi. Additionally, the heterozygous Bt rice straw extract at 20 g·L^−1^ significantly increased the aboveground dry weight, while the heterozygous Bt rice straw extract at 10 g·L^−1^ significantly inhibited both the aboveground and underground dry weights of pakchoi.

## 3. Discussion

Currently, research on the effects of Bt rice straw extract is limited. This study explored the impacts of homozygous and heterozygous Bt rice straw extracts on seed germination, protective enzyme activity, and plant growth, providing a theoretical basis for further investigating Bt rice extract effect on winter vegetables. Returning straw to the field plays a crucial role in improving soil organic matter and structure. While numerous studies highlight its benefits, potential negative effects should not be ignored. Stress factors can disrupt active oxygen metabolism, ultimately affecting plant health [43]. Many studies have shown that seed germination and seedling growth are inhibited in a concentration-dependent manner as the concentration of straw extract increases [44,45,46,47]. For instance, water extracts from the roots, stems, leaves, and tassels of corn stalks significantly inhibited the seed germination of shepherd’s purse, and the germination rate and index decreased as extract concentration increased [48]. Similarly, wheat straw extracts strongly inhibit lettuce and cress root growth, with higher concentrations associated with greater inhibition [35]. The impact of rice straw extract on sweet corn seeds and wheat germination and seedling growth was generally characterized by mild promotion at low concentrations and strong inhibition at high concentrations [49,50]. Additionally, corn straw extract has been shown to inhibit wheat and cucumber seed germination, with the inhibitory effect intensifying at higher concentrations [51,52]. Furthermore, Zhang et al. found that the allelopathic compound benzoic acid inhibits Arabidopsis seedlings’ taproot elongation by reducing the size of the meristematic and elongation zones via auxin signaling [53].

In this study, pakchoi exhibited a concentration-dependent response to Bt rice straw extracts: seed germination and root length decreased with increasing extract concentrations across all treatments. Notably, high-concentration heterozygous Bt extracts significantly suppressed germination compared to non-Bt controls, whereas low concentrations of both homozygous and heterozygous Bt extracts promoted root elongation (Table 1), consistent with Liu et al. [54]. This biphasic effect may arise from subthreshold stimulation, where low-dose Bt components enhance root cell expansion without exceeding toxicity thresholds, combined with potential hormonal modulation (e.g., auxin/cytokinin rebalancing) that promotes root growth. Intriguingly, high-concentration homozygous Bt extract increased seedling height, likely through components stimulating shoot-specific cell elongation. Furthermore, while low concentrations may activate stress-primed defense responses to facilitate root development, growth inhibition at high concentrations is likely mediated by oxidative damage, as evidenced by suppressed CAT/SOD activities (Figure 1, Figure 2, Figure 3 and Figure 4).

The experimental results demonstrated that neither homozygous nor heterozygous Bt rice straw extracts significantly affected pakchoi plant height or chlorophyll content. This finding differs from the results of Elisante et al. and Jaballah et al., maybe due to the role of Bt protein in regulating metabolite release and influencing chlorophyll levels in plants [55,56]. Furthermore, a low concentration of homozygous Bt rice straw extract significantly inhibited underground dry weight, while a low concentration of heterozygous Bt rice straw extract inhibited both aboveground and underground dry weight in pakchoi (Table 2). Gong et al. reported that incorporating corn, wheat, and Jerusalem artichoke straw promoted cucumber growth to varying degrees, with effects differing based on straw variety and cucumber growth stage [57]. The differential responses observed in our study likely reflect fundamental differences in nutrient utilization strategies between cucurbitaceous (cucumber) and brassicaceous (pakchoi) species, particularly in their metabolic responsiveness to rice-derived secondary metabolites. Nutritional profiling revealed significant compositional differences between Bt and non-Bt rice straw (Table 3). Homozygous Bt straw exhibited significantly elevated phosphorus content coupled with reduced potassium levels compared to non-Bt counterparts, while heterozygous Bt materials demonstrated more pronounced potassium deficiency. The observed growth inhibition, particularly in belowground biomass, may be associated with potassium limitation, given its critical roles in osmoregulation and nutrient translocation. However, the precise mechanistic linkage between straw nutritional characteristics and seedling growth responses warrants further investigation, particularly concerning potential interactions between Bt proteins and mineral nutrient availability.

SOD serves as the first line of defense against oxidative stress in plants and is present in every cell. Its primary function is to convert or break down toxic superoxide anions (O_2_^−^) into hydrogen peroxide (H_2_O_2_) and molecular oxygen (O_2_) [58]. CAT, an essential heme enzyme, plays a crucial role in converting hydrogen peroxide into water and oxygen, contributing to plant metabolism and signal recognition [59]. POD is a key enzyme in lignin biosynthesis, facilitating the polymerization of lignin monomers to reinforce cell structure [60]. Zhao et al. suggested that changes in antioxidant enzyme activity may result from disruptions in intracellular reactive oxygen species (ROS) metabolism caused by endogenous inhibitory substances [61]. These disruptions lead to ROS accumulation and trigger the plant’s antioxidant enzyme stress response, which can directly inhibit CAT, SOD, and POD activities in certain recipient plants. The upregulation of SOD and POD activities helps eliminate excess ROS and protect plant cells from oxidative damage. Tang et al. reported that low concentrations of straw decomposition solution had minimal effects on the antioxidant enzyme activity of common buckwheat seedlings [36]. However, as the concentration increased, SOD and POD activities significantly declined, while malondialdehyde (MDA) levels rose sharply, indicating oxidative stress-induced damage and inhibited seedling growth. Similarly, Li et al. found that high concentrations of rice stem extract increased SOD and CAT activities in germinated wheat seeds 48 h after sowing [41]. Shen et al. observed that low concentrations of rice extract could enhance CAT and POD activities in sweet corn leaves [49].

The results of this study demonstrated that, compared with the liquid phase of non-Bt rice straw extract, a high concentration of homozygous Bt rice straw extract inhibited CAT and SOD activity in pakchoi leaves and CAT activity in the roots, while promoting POD activity in the leaves (Figure 2, Figure 3, Figure 4 and Figure 5). A low concentration of homozygous Bt rice straw extract inhibited CAT and POD activity in pakchoi leaves (Figure 3 and Figure 5). Additionally, a high concentration of heterozygous Bt rice straw extract suppressed SOD activity in pakchoi leaves but enhanced POD activity in both the leaves and roots (Figure 1, Figure 5 and Figure 6). In contrast, a low concentration of heterozygous Bt rice straw extract inhibited POD activity in the leaves while promoting it in the roots (Figure 5 and Figure 6). The effects of Bt rice straw extract on the activity of protective enzymes in the roots and leaves of pakchoi were complex and varied depending on concentration and measured parameters. Chen et al. found that the antioxidant enzyme activity of maize, soybean, and wheat treated with different concentrations of maize straw extract exhibited a dual effect, either inhibiting or promoting activity [62]. Furthermore, as concentration varied, the effects did not follow a linear pattern but rather displayed diverse responses, including promotion, inhibition, alternating promotion/inhibition, or no significant change. The protective enzyme system plays a critical role in plant stress responses and growth regulation. The disruption of these protective enzyme activities reflects an imbalance in plant reactive oxygen species metabolism, suggesting that, when exposed to Bt rice straw extracts, pakchoi cells are vulnerable to oxidative damage. As a primary producer in the ecosystem, alterations in pakchoi’s physiological state can cascade through the food chain, thereby threatening the biodiversity and ecological equilibrium of the entire farmland ecosystem. These irregular changes can be attributed to the complex composition of the extracts and the inherent differences in pakchoi’s physiological regulation mechanisms. This complexity exacerbates the challenge of conducting accurate ecological risk assessments. In real-world farmland ecosystems, the diverse growth stages of pakchoi, coupled with fluctuating environmental conditions, render the ecological impacts of Bt rice straw extracts even more unpredictable. This unpredictability poses significant uncertainties in maintaining ecosystem stability.

The findings of this study provide a theoretical basis for understanding the effects of Bt rice straw extract on pakchoi seed germination, protective enzyme activity, and plant growth. However, several limitations should be acknowledged. First, the conclusions drawn here are specific to the tested Bt rice lines (homozygous and heterozygous) and the experimental conditions applied; they should not be extrapolated to other Bt crop varieties or field environments without further validation. Second, while our experiments included biological replicates, the potential interactions between Bt protein and other allelopathic compounds in straw extracts require more targeted investigations (e.g., Bt protein quantification, genetic background controls) to establish causality. Third, the short-term laboratory observations may not fully reflect the complex dynamics of straw decomposition and plant–microbe interactions in natural agroecosystems. Future research should incorporate field trials to explore the long-term impact of Bt rice straw on vegetable growth and soil ecosystems. Such studies will offer more comprehensive insights and theoretical support for optimizing Bt rice cultivation and the sustainable utilization of straw in agricultural practices.

## 4. Materials and Methods

### 4.1. Test Materials

The straw varieties used in this experiment included homozygous Bt rice (T775), heterozygous Bt rice (F1 of T775), and non-Bt conventional rice (Tianyouhuazhan). We obtained all materials from Researcher Wang Feng (Rice Research Institute of Guangdong Academy of Agricultural Sciences, Guangzhou, China). The straw materials used in this study were all sourced from field cultivation within the same experimental station. The straw samples were randomly collected from each plot and then mixed. Both homozygous and heterozygous Bt rice lines in this study express the *Cry1Ab* insecticidal protein. The target organisms of this Bt protein are mainly Lepidoptera pests such as *Chilo suppressalis* (striped stem borer) and *Tryporyza incertulas* (yellow stem borer). After the rice plants matured, the stalks (including stems and leaves) of the three varieties were collected, dried, crushed, and thoroughly mixed for subsequent use. The tested vegetable variety was pakchoi, with seeds purchased from Guangzhou Xiangsheng Seed Co., Ltd. (Guangzhou, China).

### 4.2. Preparation of Extract

Straw samples of homozygous Bt rice, heterozygous Bt rice, and non-Bt rice were each prepared from a composite of stems and leaves randomly collected from 20 healthy field-grown plants per variety. After oven-drying at 65 °C (DHG-9140A drying oven, Shanghai Jinghong Experimental Equipment Co., Ltd., Shanghai, China) to constant weight, the materials were homogenized through a 40-mesh sieve. Precisely, 4.0 g of each processed straw sample was weighed into 100 mL conical flasks. Each flask was then filled with 100 mL of distilled water, sealed with film, and placed on a shaker (TS-2102 constant temperature oscillator, Shanghai Tiancheng Experimental Equipment Co., Ltd., Shanghai, China) at 25 °C with a rotation speed of 100 rpm for 24 h. After soaking, the mixture in each flask was transferred to a 50 mL centrifuge tube and centrifuged at 4000× *g* for 10 min at room temperature. The supernatant was collected to obtain the rice straw extract mother liquor at a concentration of 40 g·L^−1^. The physicochemical properties of the tested rice straw extracts are shown in Table 3. Finally, the mother liquor was diluted with distilled water to prepare solutions at concentrations of 10 g·L^−1^, 20 g·L^−1^, and 40 g·L^−1^, which were then stored at 4 °C for further use. Total nitrogen was determined by the Kjeldahl method, total phosphorus by the molybdenum–antimony anti-spectrophotometric method, total potassium by flame photometry, and Bt protein by double-antibody sandwich ELISA (EnviroLogix Cry1Ab/1Ac Plate Kit, EnviroLogix Inc., Portland, ME, USA).

### 4.3. Seed Germination Test

Using the Petri dish filter paper germination method, surface-sterilized pakchoi seeds (0.5% H_2_O_2_ for 15 min, followed by 2–3 rinses with deionized water) were evenly distributed on qualitative filter paper in sterile 9 cm Petri dishes (25 seeds/dish). Each treatment consisted of four replicate dishes (co-cultivation), with 5 mL of rice straw extract at specified concentrations (10, 20, or 40 g·L^−1^). The samples were incubated in an artificial climate chamber (light/dark cycle: 12/12 h, temperature: 25 °C, humidity: 70%) for one week. The number of germinated seeds was recorded, and four representative seedlings were randomly selected from each treatment group to measure root length, seedling height, and dry weight. Additionally, leaf and root samples were collected to determine the activities of SOD, POD, and CAT in the leaves and roots of pakchoi. All treatment groups were established with four independent biological replicates. For detailed sample sizes and replication strategies, refer to Supplementary Table S1.

### 4.4. Plant Growth Test

Pakchoi seedlings with uniform growth were selected and transplanted into pots containing 600 g of soil (dimensions: upper diameter × lower diameter × height = 12 cm × 9 cm × 12 cm) in a completely randomized design. Three concentrations of rice straw extract were applied, i.e., (1) 10 g·L^−1^, (2) 20 g·L^−1^, and (3) 40 g·L^−1^. The experiment included three rice varieties, i.e., homozygous Bt rice (T775), heterozygous Bt rice (F1 generation of the T775 hybrid), and non-Bt ordinary rice (Tianyouhuazhan), and four replicates were set up for each treatment. After the pakchoi seedlings reached a stable growth state, the straw extract solution was added once every two days, a 5 mL volume each time (injected into the soil around the roots). Plant height, chlorophyll content, and the dry weight of both aboveground and belowground parts were measured in the sixth week after treatment.

### 4.5. Measurement Items

The germination rate was calculated using the following formula: germination rate (%) = (number of germinated seeds ÷ total test seeds) × 100%. Seedling height and root length were measured using a vernier caliper, while the height of pakchoi plants was measured with a measuring tape. Chlorophyll content was determined and calculated following the method of Wang Xuekui [63]. The aboveground and belowground parts of pakchoi were dried in an oven at 105 °C for 30 min, then further dried at 60 °C until a constant weight was achieved, and the dry weight was recorded. SOD activity was measured using the NBT photochemical reduction method following the protocol of Gao Junfeng [64]. CAT activity was determined using the hydrogen peroxide decomposition method according to Li Hesheng [65]. POD activity was assessed using the guaiacol method, following the method described by Gao Junfeng [64].

### 4.6. Data Analysis

The data were organized and charts were created using Excel 2010. Statistical analyses were performed using SPSS 25.0 software, with one-way ANOVA conducted to assess variations among treatments. Duncan’s multiple range test was used for pairwise comparisons and significance analysis of differences.

## 5. Conclusions

There were significant differences in the effects of homozygous and heterozygous Bt rice straw extracts on seed germination, protective enzyme activity, and plant growth in pakchoi. A low concentration of homozygous Bt rice straw extract significantly promoted root elongation in pakchoi seedlings but inhibited CAT and POD activities in the leaves and reduced underground dry weight. At a high concentration, the homozygous Bt rice straw extract significantly increased seedling height while inhibiting CAT and SOD activities in the leaves. However, it also significantly enhanced POD activity in the leaves. Similarly, a low concentration of heterozygous Bt rice straw extract significantly increased root length and POD activity in pakchoi seedlings but inhibited POD activity in the leaves and reduced both aboveground and underground dry weight. At a high concentration, heterozygous Bt rice straw extract significantly suppressed seed germination and SOD activity in the leaves, while significantly enhancing POD activity in both the leaves and roots. Additionally, a 20 g·L^−1^ concentration of heterozygous Bt rice straw extract significantly promoted aboveground dry weight. In conclusion, compared to the non-Bt rice straw extract, both Bt rice straw extracts exhibited varying degrees of inhibitory and promotive effects on seed germination, protective enzyme activity, and plant growth in pakchoi, without following a clear or consistent pattern.

## Figures and Tables

**Table 1:** Effects of Bt rice straw extract on germination rate and seedling growth of pakchoi.

Treatment	Concentration (g·L^−1^)	Germination Rate (%)	Root Length (cm)	Seedling Height (cm)	Dry Weight (g)
T775	10	83 ± 3.42 a	3.1 ± 0.22 b	0.86 ± 0.01 a	2.33 ± 0.14 a
F1		86 ± 4.16 a	3.81 ± 0.25 a	0.89 ± 0.11 a	2.23 ± 0.08 a
Tianyouhuazhan		89 ± 1.92 a	3.92 ± 0.14 a	0.77 ± 0.05 a	2.21 ± 0.1 a
T775	20	77 ± 6.81 a	3.09 ± 0.51 a	1.13 ± 0.19 a	2.04 ± 0.34 a
F1		78 ± 1.16 a	4.63 ± 0.27 a	1.02 ± 0.07 a	2.53 ± 0.11 a
Tianyouhuazhan		82 ± 9.02 a	3.61 ± 0.58 a	1.02 ± 0.12 a	3.26 ± 0.53 a
T775	40	74 ± 2.58 a	2.05 ± 0.13 ab	0.88 ± 0.07 b	2.2 ± 0.14 a
F1		77 ± 1.00 a	2.95 ± 0.44 a	1.16 ± 0.04 a	2.53 ± 0.04 a
Tianyouhuazhan		56 ± 4.32 b	1.41 ± 0.21 b	0.75 ± 0.06 b	2.53 ± 0.12 a

The data in the table are presented as mean ± standard error. Values within the same column sharing the same letter indicate no significant difference (*p* > 0.05), whereas different letters denote a significant difference (*p* < 0.05). “T775” refers to the straw of non-Bt rice; “F1” refers to the straw of homozygous Bt rice; “Tianyouhuazhan” refers to the straw of heterozygous Bt rice.

**Table 2:** Effects of Bt rice straw extracts on growth of pakchoi.

Treatment	Concentration (g·L^−1^)	Plant Height (cm)	Chlorophyll (mg·g^−1^)	Aboveground Dry Weight (g)	Underground Dry Weight (g)
T775	10	30.16 ± 1.38 a	1.12 ± 0.21 a	5.48 ± 0.49 a	0.34 ± 0.04 a
F1		30.15 ± 1.41 a	1.37 ± 0.09 a	4.45 ± 0.43 ab	0.23 ± 0.01 b
Tianyouhuazhan		27.2 ± 1.2 a	1.06 ± 0.06 a	4.11 ± 0.3 b	0.21 ± 0.02 b
T775	20	28.41 ± 1.89 ab	1.03 ± 0.18 a	4.02 ± 0.71 b	0.34 ± 0.18 a
F1		26.95 ± 1.05 b	1.2 ± 0.04 a	4.35 ± 0.42 ab	0.23 ± 0.04 a
Tianyouhuazhan		31.15 ± 1.08 a	1.18 ± 0.12 a	5.92 ± 0.62 a	0.25 ± 0.03 a
T775	40	29.38 ± 1.9 a	1.06 ± 0.12 a	4.48 ± 0.84 a	0.19 ± 0.03 a
F1		27.89 ± 1.91 a	1.24 ± 0.12 a	4.34 ± 0.49 a	0.18 ± 0.03 a
Tianyouhuazhan		29.84 ± 0.63 a	1.53 ± 0.17 a	5.04 ± 0.21 a	0.25 ± 0.03 a

The data in the table are presented as mean ± standard error. Values within the same column sharing the same letter indicate no significant difference (*p* > 0.05), whereas different letters denote a significant difference (*p* < 0.05). “T775” refers to the straw of non-Bt rice; “F1” refers to the straw of homozygous Bt rice; “Tianyouhuazhan” refers to the straw of heterozygous Bt rice.

**Table 3:** Physicochemical properties of rice straw extracts.

Index	Non-Bt	Homozygous Bt	Heterozygous Bt
Bt Protein (ng·g^−1^)	=LOD	1405.21 ± 77.21 a	1159.42 ± 38.97 b
Total Nitrogen (mg·L^−1^)	5.58 ± 0.50 a	4.29 ± 0.50 a	5.01 ± 0.29 a
Total Phosphorus (mg·L^−1^)	15.10 ± 0.65 b	25.52 ± 1.86 a	13.14 ± 1.02 b
Total Potassium (mg·L^−1^)	71.19 ± 1.14 a	24.62 ± 0.64 c	31.46 ± 0.22 b

The data in the table represent mean values ± standard error (SE). Different lowercase letters within the same row indicate significant differences according to Duncan’s multiple range test (*p* < 0.05). LOD means below the limit of detection (0.01 mg/L).

## Data Availability

The data presented in this study are available on request from the corresponding author.

## Supplementary Materials

The following supporting information can be downloaded at: https://www.mdpi.com/article/10.3390/plants14121797/s1, Table S1: Experimental treatment group design and replication details.
