# Supplementary material for: Effects of Bt Rice Straw Extract on Seed Germination and Plant Growth of Pakchoi: Novel Variables of Cropping System
Source: Plants (Basel). 2025 Jun 12;14(12):1797. doi: 10.3390/plants14121797 (PMC12196660; doi:10.3390/plants14121797)
Supplement: Supplementary file 1 [file plants-14-01797-s001.zip › plants-3593491-supplementary.pdf]

Table S1. Experimental treatment group design and replication details

Treatment	Extract Concentration ($g \cdot L^{-1}$)	Biological Replicates	Technical Replicates (Samples/Replicate)	Total Sample Size (n = Biological × Technical)
Non-Bt	10<br>20<br>40	4	25 seeds/dish, 4 seedlings measured	4×25 seeds, 4×4 seedlings
Homozygous Bt	10<br>20<br>40	4	25 seeds/dish, 4 seedlings measured	4×25 seeds, 4×4 seedlings
Heterozygous Bt	10<br>20<br>40	4	25 seeds/dish, 4 seedlings measured	4×25 seeds, 4×4 seedlings
